# Do Professionals Take Over? Professionalisation and Membership Influence in Civil Society Organisations

**DOI:** 10.1007/s11266-020-00214-9

**Published:** 2020-03-24

**Authors:** Frederik Heylen, Evelien Willems, Jan Beyers

**Affiliations:** Department of Political Science, University of Antwerp, Antwerp, Belgium

**Keywords:** Civil society organisations, Professionalisation, Membership influence, Political insiders

## Abstract

While many scholars have postulated the decline of membership influence as an important consequence of the professionalisation of civil society organisations (CSOs), other analysts have argued that traditional membership-driven CSOs are resilient and that hiring professionals does not necessarily diminish membership influence. This study sheds light on this issue by analysing membership influence in a representative sample of approximately 2000 CSOs from  five European countries and the European level. As members generally have a strong influence on CSOs’ policy positions, our analysis demonstrates that the pessimistic tone in much contemporary scholarly work is largely unwarranted. On the contrary, hiring professionals does not invariably decrease membership influence and can, when members are closely engaged in advocacy work, even facilitate it.

**Electronic supplementary material:**

The online version of this article (10.1007/s11266-020-00214-9) contains supplementary material, which is available to authorized users.

## Introduction

The influence members exert on the advocacy activities of civil society organisations (CSOs) varies considerably. In some CSOs, members are a vital part of the organisational apparatus, as no policy positions can be adopted without their approval. In other CSOs, advocacy work is largely decided upon and carried out by professional staff (Binderkrantz 2009, p. 668). In the past decades, scholars—especially those in Anglo-Saxon countries—have reported on the proliferation of professionalised groups lacking a traditional structure of local branches and/or internal processes of membership engagement and instead relying on loosely affiliated financial supporters. CSOs have become more ‘business-like’; they have hired more full-time paid staff and have adopted various market-based management practices (Eikenberry and Kluver 2004; Hwang and Powell 2009; Maier et al. 2016). One prominent concern is that when CSOs reach a certain size, professionals take over, and the status of members is marginalised to that of ‘clients’, ‘check book participants’, ‘credit card members’ or ‘cash cow supporters’ (Hwang and Powell 2009; Jordan and Maloney 1997, 2007; Maier et al. 2016; McCarthy and Zald 1973; Rothenberg 1992; Skocpol 2004). When seeking to influence public policy, these CSOs often do not need to rely on their supporters for consultation and endorsement; rather, they develop expertise and carry out advocacy work by means of professional staff.

More recent research, however, points to a different reality in which both professionalised CSOs without closely involved members or supporters, and professionalised CSOs with extensive membership engagement structures and procedures coexist (Albareda 2018; Albareda and Braun 2019; Grömping and Halpin 2019; Minkoff and Powell 2006; Walker et al. 2011). This is puzzling as a common presumption is that CSOs with engaged members lack the flexibility and strategic direction to act as meaningful long-term government interlocutors (Greenwood 2002, p. 65; Schmitter and Streeck 1999, p. 50). Involving members and supporters in advocacy work is often considered to be time-consuming and costly, while expertise is deemed to be more efficiently developed by professionals (Grömping and Halpin 2019; Hwang and Powell 2009). Consequently, professionalisation is often perceived as beneficial for gaining policy access and influence, while strongly engaged and influential members are not. Hence, the question emerges of how to explain the persistence of membership-based CSOs and their varying degrees of membership influence.

Hinting at one possible explanation, new research demonstrates that the influence members can wield over political positioning might actually help CSOs to gain access to the policymaking process, precisely because of their engaged membership base that serves as a source of political support. It is then the prospect of gaining access and influence that incentivises CSOs to maintain and foster membership involvement structures and procedures (Albareda and Braun 2019; Grömping and Halpin 2019). Additionally, giving a voice to members is vital for CSOs to prevent ‘organisational detachment’, which could threaten their survival (Knoke 1981, pp. 154–155).

Although members can influence many aspects of CSOs, this article focuses on members’ influence on political positioning, i.e. the degree to which members can shape CSOs’ positions on specific public policies. This focus springs from the presumed tension between professionalisation and membership influence as challenging the democratic role that CSOs can play (Hwang and Powell 2009; Maier et al. 2016; Skocpol 2004). The classic view is that by closely involving members in the establishment of political positions, CSOs function as intermediaries between society and political elites (Albareda 2018; Halpin 2006; Jordan and Maloney 1997, 2007). CSOs acting on behalf of their members and supporters, after being endorsed via internal consultations that make the leadership accountable to those members, demonstrate their ‘representativeness’ (Halpin 2006; Hollman 2018; Johansson and Lee 2014). Hence, the proliferation of professionalised CSOs without membership engagement may have troubling consequences for democratic governance since these organisations’ policy views are only loosely connected to the preferences of their members and broader societal segments (Eikenberry and Kluver 2004; Jordan and Maloney 1997; Maier et al. 2016; Skocpol 2004; Walker et al. 2011).

In developing a conceptual framework to explain the degrees of membership influence in CSOs, we draw on a rich scholarly tradition that dates back to Michels’ so-called ‘iron law of oligarchy’ (1915), which states that power is concentrated within a small elite of organisational leaders. Michels’ work has influenced research on social movement organisations, interest groups and third-sector organisations—which we subsume under the overarching term of ‘civil society organisations’. Although scholars studying CSOs have identified external environmental constraints (e.g. a strong involvement in public policymaking and/or dependency on government funding) and internal organisational factors (e.g. the CSO’s internal structure and/or staff) as factors which may affect membership influence, these variables and their relative importance have rarely been systematically tested in large-*N* comparative studies (e.g. Eikenberry and Kluver 2004; Jordan and Maloney 2007; McCarthy and Zald 1973). Much of what we know about membership influence is based on case studies or scant empirical evidence on one specific type of CSO (for an exception, see Binderkrantz 2009). Consequently, we simply do not know whether and how membership influence is associated with CSOs employing professionals or being strongly involved in public policymaking.

The empirical corpus of the paper presents survey evidence collected from more than 2000 CSOs across six European polities, namely Belgium, the Netherlands, Slovenia, Lithuania, Sweden and the European Union (EU). Instead of focussing on a narrow set of CSOs, we analyse the impact of external environmental constraints and internal organisational factors for a broad range of CSOs (e.g. both citizen groups and business associations). The analysis demonstrates that CSOs employing professionals are associated with higher levels of membership influence, contingent upon them also having high degrees of membership involvement. As such, employing professionals might be beneficial for membership influence, which in turn may strengthen the political status of CSOs relative to policymakers. Additionally, the findings demonstrate that policy insiders display higher levels of membership influence. The results therefore nuance—and to a certain extent even qualify—the iron law of oligarchy and shed light on one of the most important concerns in the interest representation literature (Diefenbach 2019).

## Professionalisation: Constraining or Facilitating Membership Influence?

An important concern in much contemporary scholarship is that when CSOs reach a certain size, professionals take over, with the status of members then reduced to that of financial donors without much influence within the organisation (Jordan and Maloney 1997; Maier et al. 2016; McCarthy and Zald 1973; Rothenberg 1992; Skocpol 2004). Much of this claim resonates with Michels, who argued that limited membership influence is associated with the ‘elitisation’ of organisational leaders mostly acting in their own interests (1915, p. 76). According to Michels, due to ‘practical and technical necessity’, the very notion of an organisation implies a concentration of power at the top, which comes at the cost of ‘rank and file’ members; therefore, ‘Every party or professional union becomes divided into a minority of directors and a majority of directed’ (1915, p. 72).

Six decades after Michels proposed his iron law of oligarchy, McCarthy and Zald stated that as social movement organisations ‘become routinised and oligarchic, leaders become more and more distant from the group whose interest they presumably represent’ (1973, p. 13). This observation continues to be reflected by contemporary scholars positing that as CSOs become more professionalised, the influence of their members declines (Eikenberry and Kluver 2004; Jordan and Maloney 1997, 2007; Maier et al. 2016; Skocpol 2004). The undertone in much of this literature is somewhat pessimistic as it deplores that professionalised CSOs can only limitedly act as intermediaries connecting their members and broader societal segments with political elites. CSOs experience deeply ingrained tension between professionalisation and membership influence, putting pressure on their function as collective representatives.

This tension between professionalisation and membership influence is inexorably tied to the governability of CSOs and is notably captured in Schmitter and Streeck’s characterisation of the ‘logic of influence’ and the ‘logic of membership’ (1999, p. 19; see also Bennett 2000; Greenwood 2002; Grömping and Halpin 2019; Kohler-Koch and Buth 2013). The organisational leadership must strike an appropriate balance between acting on members’ demands and what the CSO must do to ensure its survival and policymaking success. Many scholars have contended that a trade-off exists between these two logics, and, taken to the extreme, both logics may inhibit organisational functioning (Jordan and Maloney 1997, 2007; Kohler-Koch and Buth 2013; Skocpol 2004; van Deth and Maloney 2012).

On the one hand, CSOs can choose to hire professional staff with a specific educational or training background (e.g. judicial, research), to monitor policies, to develop and manage organisational objectives and strategies, and network with other stakeholders in order to meet policymakers’ demands for expertise (Halpin and Fraussen 2017; Hwang and Powell 2009). In return for this policy expertise, policymakers may provide access to or they may subsidise CSOs’ projects (Albareda and Braun 2019; Eikenberry and Kluver 2004; Maier et al. 2016). However, it is often presumed that professionalisation and involvement in public policymaking, combined with shifted resource dependencies, could lead to declining levels of membership influence. For instance, membership-based CSOs that heavily depend on government funding may moderate or even alter their advocacy work to comply with government priorities and regulations attached to that funding, to the detriment of members’ interests (Bloodgood and Tremblay-Boire 2016; Mosley 2012; Neumayr et al. 2015). And, as argued by Knoke (1981, pp. 154–155), when members experience the inability to exercise influence within the organisation, the likelihood of ‘organisational detachment’ increases, threatening the very existence of the organisation.

On the other hand, organisations that are too closely controlled by their members might lack the flexibility and strategic direction needed to act as long-term government interlocutors (Greenwood 2002, p. 65; Schmitter and Streeck 1999, p. 50). Close engagement with members might impede CSOs to accommodate policymakers’ political demands. Membership involvement is time-consuming and costly, decreasing organisational flexibility to adapt and react to changing political contexts (Grömping and Halpin 2019; Hollman 2018; van Deth and Maloney 2012; Walker et al. 2011). Close consultation with members and supporters might also give rise to internal disagreement; hence, CSOs that do not want to alienate their members and will avoid to take action on policies their members do not support (Grömping and Halpin 2019; Minkoff and Powell 2006; Strolovitch 2006). As such, CSOs that invest in membership involvement and voluntary activities are often believed to be less directly involved in policymaking processes (Kohler-Koch and Buth 2013; Skocpol 2004). In short, it is often presumed that CSOs cannot do well by policymakers, while simultaneously doing good for their members.

To balance these two needs—namely, involving members in internal processes (i.e. the logic of membership) and achieving political goals (i.e. the logic of influence)—CSOs can adopt varying organisational structures and procedures. However, we expect that professionalisation does not necessarily conflict with membership influence. CSOs can simultaneously hire professionals to develop and supply expertise to policymakers while adopting organisational structures and procedures to enable member participation. Recent research has underscored this assertion. Many professionalised CSOs feature extensive structures and procedures for membership involvement (Albareda 2018; Albareda and Braun 2019; Grömping and Halpin 2019; Walker et al. 2011). Moreover, this research demonstrates that the proliferation of professionalised and staff-led CSOs is less prevalent than asserted by scholars in the tradition of Skocpol’s historical analysis of the increase in professional but ‘memberless’ CSOs (2004; Skocpol et al. 2000, Jordan and Maloney 1997, 2007; Minkoff and Powell 2006). This leads us to ask how varying degrees of membership influence relate to professionalisation.

While much research has addressed the drivers of professionalisation and its effects on CSO community-building, service-delivery and advocacy work (see Eikenberry and Kluver 2004; Maier et al. 2016), research analysing the factors that affect membership influence within organisations is scarce. Nonetheless, some scholars point at factors that potentially increase membership influence (Albareda 2018; Halpin 2006; Johansson and Lee 2014; Maier and Meyer 2011). Broadly speaking, they emphasise the importance of internal structures and organisational processes; more precisely, the extent to which members can speak up and have a voice within CSOs. Organisational capacity—namely, the presence of professionals and a differentiated organisational structure—is considered vital to facilitating internal processes of membership involvement and influence. For example, professionals can organise internal elections for the board at the general assembly, support task-specific working groups, and initiate membership consultations. Often these tasks constitute a full-time job and hiring staff to fulfil these tasks can thus strengthen CSOs in their engagement with the membership. Moreover, by involving members in political positioning, such CSOs demonstrate their ‘representativeness’ to policymakers and can bring members’ active support to the policy table.

These CSOs can ensure policymakers that they rely on internal alignment and consensus (Albareda and Braun 2019; Grömping and Halpin 2019; Hollman 2018). Hence, despite these internal processes being time-consuming and costly, many CSOs maintain such organisational procedures.

We contribute to this burgeoning debate on how professionals affect membership influence by focussing on both internal organisational factors and external environmental factors. As the above discussion makes clear, how and to what extent professionals interact and engage with members may crucially affect the influence those members exert on CSOs’ political positioning. We further examine how membership influence is constrained or facilitated by external factors linked to the logic of influence. We expected the extent to which CSOs are part of the innermost political circles and the extent to which they depend on government funding—two frequently examined factors in the literature—to affect membership influence. Access to the policymaking process and government funding can be vital for achieving organisational goals such as political influence and organisational maintenance.

## Hypotheses

This section further disentangles the relation between professionalisation and membership influence and then proposes hypotheses on internal organisational pressures and external dependencies. First, we conceive professionalised organisations as having a professional staff and a differentiated organisational structure. Given the complex legal and institutional environment in which CSOs operate, professionals lead many CSOs and engage in political advocacy in their name. Compared to rank-and-file members, professionals are presumed to be more committed to their field of expertise and/or to peers in their field (Hwang and Powell 2009; Jordan and Maloney 2007; Staggenborg 1988). In this regard, the criteria for hiring staff are expertise, experience and connections, and a strong commitment to a particular cause is not always a requirement. Hence, hiring professionals can result in CSOs that are less aligned with the grievances and needs of their members (Eikenberry and Kluver 2004; Jordan and Maloney 2007; Kohler-Koch and Buth 2013; Kreutzer and Jäger 2011; Maier et al. 2016; Sanders and McClellan 2014). Specifically, professionals whose livelihoods are intertwined with organisational survival might be more predisposed to focus on issues of organisational maintenance than on informing or involving the membership; this scenario can potentially lead to ‘mission drift’ or ‘goal displacement’. In addition, professionals often favour a business-like style of management in which they refer to their members as ‘customers’ or ‘clients’, which can alienate the membership. Hiring professional staff may thus result in less membership influence.

Professionalisation is not solely a matter of hiring professional staff; CSOs also vary in the organisational structures they adopt to develop expertise—in other words, how they gather, process and disseminate information. CSOs make decisions on how staff and other human resources (e.g. members and volunteers) are deployed. One important aspect is functional differentiation, which concerns the extent to which CSOs have separate units, committees or departments dealing with specific tasks (Albareda 2018; Albareda and Braun 2019, p. 471). For instance, while some CSOs invest in internal research units dedicated to specific issues, others establish units concerned with conducting public advocacy campaigns, coordinating volunteer activities or recruiting sponsors. Hence, CSOs that have created units with policy specialists and/or campaign professionals can strengthen their advocacy work. Functional differentiation thus reflects organisational choices and priorities. While such task-specific units may lead to fewer internal coordination difficulties, they could also increase transaction costs (Hollman 2018). Though, as CSOs are functionally differentiated, members might lack the necessary time and skills to control all organisational activities (Michels 1915; Jordan and Maloney 2007; van Deth and Maloney 2012). Our expectations on the effects of staff size and functional differentiation are summarised in Hypothesis 1: *The more professionalised CSOs are, the lower the membership influence.*

Nevertheless, how staff size and internal differentiation can affect membership influence might be moderated by the overall degree of membership involvement. Membership involvement denotes a general propensity to engage members in various internal processes—such as meeting in working groups to discuss policy objectives, selecting the organisational leadership and developing and executing advocacy strategies—allowing members to endorse group representatives and hold them accountable (Albareda 2018; Halpin 2006; Johansson and Lee 2014; Knoke 1981). As such, the concept of membership involvement goes beyond the extent to which members decide on the CSO’s political positions.

Although Michels (1915) and more recent authors (e.g. Jordan and Maloney 2007; van Deth and Maloney 2012) have assumed that members are unable or unwilling to become involved in CSOs, the actual extent of membership involvement might vary considerably. Salisbury (1969) pointed out that many members and supporters of CSOs are not interested in passive involvement and want to express themselves when policy positions are formulated. Many members and supporters wish to be actively involved because they feel a strong commitment to the cause—such as peace, human rights, environmental protection—that CSOs are defending, while in other CSOs the economic self-interest of members is at stake, which sparks a wish to be closely engaged (De Bruycker et al. 2019).

In this regard, the impact of professionalisation on membership influence might depend on the degree of overall membership involvement. On the one hand, when members are limitedly involved in organisational processes, employing more staff may simply reinforce an existing pattern and diminish membership influence. When staff are granted more discretion, they can become more influential at the expense of members (Kreutzer and Jäger 2011). These members might even see delegating complicated tasks such as formulating policy positions to professionals as an attractive organisational quality (Jordan and Maloney 2007). On the other hand, more engaged members might limit the staff’s discretion and outsource well-delineated supportive tasks. CSOs can also establish checks and balances in their internal decision-making procedures and adopt formal electoral mechanisms to appoint board members and select the leadership (Maier and Meyer 2011). Functional differentiation not only allows CSOs to produce expertise but also creates venues through which members can become involved in internal processes. Such differentiation makes internal processes more predictable and can facilitate an internal structure for consultation and deliberation (Albareda 2018; Hollman 2018). In this regard, the hiring of more staff and internal differentiation may facilitate and strengthen membership influence. Tasks such as answering members’ emails, organising working groups and general assemblies and aggregating members’ concerns may constitute a full-time occupation and depend on smooth internal procedures, while members can use the CSO’s dedicated venues to discuss policy positions and reach internal alignment (Hollman 2018). Our expectations are presented in Hypothesis 2: *The effect of professionalisation (staff size and functional differentiation) varies according to the degree of membership involvement. At low degrees of involvement, professionalisation decreases membership influence. At high degrees of involvement, professionalisation increases membership influence.*

From internal determinants, we turn to the dependencies CSOs experience vis-à-vis the external environment. We first focus on how political insiderness affects membership influence. ‘Political insiderness’ is the degree to which CSOs regularly participate in policymaking processes, gain access to policymakers and political institutions, and enjoy recognition as government interlocutors (Fraussen et al. 2015; Halpin and Fraussen 2017; Maloney et al. 1994). For Schmitter and Streeck (1999, p. 19), organisational leaders must balance acting on the will of their members (*logic of membership*) and broader organisational goals, such as becoming a long-term governance partner or shaping public policy (*logic of influence*). CSOs becoming more enmeshed with the political environment could ensure organisational legitimacy and maintenance, but such enmeshment can also disempower members. If CSOs become regular government interlocutors, they need sufficient autonomy from the membership to react flexibly to policymakers’ demands (Greenwood 2002, p. 65); close membership involvement in internal decision-making processes may slow CSOs’ reactivity to changing political contexts (Hollman 2018). Indeed, to allow for political manoeuvring, the organisational leadership needs a certain degree of autonomy from the membership (Beyers 2008, p. 1203), and such autonomy may conflict with an overly influential membership body that imposes immediate demands and short-term interest perceptions on the CSO (Greenwood 2002). This leads to Hypothesis 3: *The higher the degree of CSOs’ political insiderness, the lower the degree of membership influence.*

Closely tied to this political environment are the funding streams CSOs need to survive. McCarthy and Zald have stated that ‘outside financial support’, such as government funding, ‘means that a membership in the classical sense is almost dispensable’ (1973, p. 18). In this regard, decreased reliance on the membership for resources is an important factor predicting lower levels of membership influence. This mechanism is best understood in the framework of resource dependency theory, which states that to survive and reach their goals, organisations need external resources, which consequently makes them sensitive to their donors’ preferences (Pfeffer and Salancik 1978; see also Bloodgood and Tremblay-Boire 2016; Mosley 2012; Neumayr et al. 2015). When resources mainly come from the membership, members have an important lever with which they can control the organisation. However, to gain organisational autonomy, the leadership may seek to diversify their resource dependencies, for instance, by seeking government funding (Eikenberry and Kluver 2004; Froelich 1999). When funding is increasingly obtained from outside the membership base, CSOs become less dependent on members and this reduces the extent to which members can control the CSOs’ political positioning. In the words of McCarthy and Zald, ‘the donor to such a movement has little control over the movement leadership short of withholding funds’ (1973, p. 18). Moreover, the rules and requirements attached to government funding impact CSO advocacy, possibly to the detriment of members’ interests (Bloodgood and Tremblay-Boire 2016; Mosley 2012; Neumayr et al. 2015). Hence, we propose Hypothesis 4: *The more dependent CSOs are on government funding, the lower the degree of membership influence.*

## Research Design

The data analysed are part of a project that surveyed CSOs in various European countries  (Beyers et al. 2020; www.cigsurvey.eu). The survey focussed on topics such as political strategies, organisational development and management. The analyses relied on evidence from Western (Belgium and the Netherlands), Eastern (Slovenia and Lithuania) and Scandinavian (Sweden) countries and from the EU-level. These systems vary in how state-society relations are institutionalised. One way to characterise interest representation systems is by placing them on a pluralist–corporatist continuum. For instance, Belgium and Sweden are often characterised as neo-corporatist, while Lithuania can be described as rather pluralistic (Jahn 2016; Siaroff 1999). According to Streeck, the logic of influence corresponds with neo-corporatist arrangements in which CSOs are granted ‘organisational security, e.g. by guaranteeing them privileged access and recognising them as the sole representative’ (1983, p. 266). In contrast, in more pluralistic settings, CSOs are less able to rely on the state for resources; thus, the logic of membership is more likely to prevail (Schmitter and Streeck 1999).

This paper focusses on membership organisations, which are organised entities that aggregate and represent the political interests of their members and supporters before the government and/or the broader public. We conceptualise membership organisations as CSOs that encompasses the wide array of entities representing the organisational fabric between society and the government. Hence, our sample includes CSOs representing the interests of citizens, such as labour unions and women’s organisations, and CSOs representing public causes, such as environmental associations. It also includes CSOs representing business interests, such as associations of construction companies and specific occupations (e.g. hospital nurses). In this regard, membership denotes formally registered members as well as loosely affiliated supporters who donate time and/or financial resources. We expected the tension between members’ interests and professionals to be most pronounced in CSOs with a traditional membership base. That said, similar to Jordan and Maloney (2007), we also included CSOs with so-called ‘supporters’ because (1) supporter-based organisations also experience a tension between their supporters’ interests and what is necessary for the CSO to influence public policy. Additionally, (2) in many instances, CSOs have incentives to engage their supporters in crafting policy positions because the exit of supporters may threaten organisational survival. For instance, a CSO like Amnesty International, which is strongly staff-led and reliant on informal supporters, invests considerably in establishing and engaging local groups which carry out advocacy work.

Furthermore, comparing business associations (and professional associations) with other organisation types is fruitful for our research purposes. Business associations are often associated with high levels of professionalisation—having abundant financial resources and staff—while they are also dependent on their corporate members for funding and technical expertise. Moreover, as their economic self-interest is at stake, members of business associations often wish to be closely involved in political positioning (De Bruycker et al. 2019; Halpin 2006). Therefore, business associations were expected to face strong incentives to closely engage with their members, resulting in significant membership influence on policy positions. Citizen groups were conversely presumed to be less induced to closely engage with their members. The supporters of these CSOs are often not the direct beneficiaries of the organisations’ advocacy work because citizen groups defend particular public causes (e.g. climate change, traffic safety) or disenfranchised societal segments (e.g. the poor, children, animals) beyond their members’ self-interest (Halpin 2006); consequently, these CSOs find it somewhat easier to position themselves on concrete policy dossiers (De Bruycker et al. 2019). In these CSOs, ‘fidelity to mission is critical in order to sustain participation’ (Minkoff and Powell 2006, p. 593), while the authorisation and mandate to engage in advocacy work do not necessarily need to come directly from supporters since their individual self-interest is less directly at stake (Halpin 2006).

Our analyses focussed on CSOs mobilising nationwide constituencies and excluded associations mobilised at the local level (e.g. provinces and cities). The sampling procedures applied in the six political systems identified 9850 CSOs, of which 3732 CSOs completed the survey (average response rate = 38.5%). After omitting missing values across variables, we had a final dataset including 2061 CSOs. The Appendix provides further details on the sampling procedures and fieldwork (see also Beyers et al. 2020).

To measure membership influence, the dependent variable, we asked respondents the following question: ‘Thinking about your organisation’s position on public policies, how would you rate the influence of your membership?*’* Respondents could rate membership influence on a five-point Likert scale ranging from ‘very influential’ to ‘not at all influential’. In total, at 41% of the CSOs, members were ‘very influential’, and 43% of the respondents indicated that their members were ‘moderately influential’. Only 13% of the respondents reported that members were ‘somewhat influential’ and 3% selected ‘not at all influential’. As such, the two low-influence categories were collapsed, which resulted in an ordinal variable consisting of three categories.

To gauge professionalisation, we created two variables. First, professional staff was measured as the logarithmically transformed number of employees; the log transformation resulted in a minimum of − 2.3, a mean of 0.87 (*α* = 1.94) and a maximum of 9.68. Second, to measure functional differentiation, we used the same measurement as Albareda (2018, p. 7), namely whether the CSO has established committees for specific tasks; 1382 of the responding CSOs (67%) had such committees.

To measure membership involvement, we created an index based on the following question: *‘*How important are your members for the following activities?’ These activities were: (1) ‘helping to influence public policy’, (2) ‘providing ideas about your organisation’s campaigning strategies’, (3) ‘identifying problems or providing ideas about your organisation’s activities’ and (4) ‘providing evidence of support from affected members or concerned citizens’. Respondents could indicate the intensity of membership involvement on a five-point Likert scale ranging from ‘unimportant’ to ‘very important’. These responses were then summarised to create a scale ranging from 0 to 20 with a mean of 16.2 (*α* = 3.21) and a polychoric ordinal alpha of 0.70. An alternative measure capturing whether members, the board or professional staff decide on political positions is operationalised in the Appendix.

For assessing political insiderness, we established a five-point Likert scale (ranging from ‘never’ to ‘once a week’) exploring the frequency at which CSOs respond to open consultations’, ‘served on advisory commissions/boards’ and ‘presented research results or technical information to policymakers’. This scale ranged from 0 (three times ‘never’) to 12 (three times ‘once a week’) with a mean of 3.23 (*α* = 2.45) and a polychoric ordinal alpha of 0.85. Finally, for measuring dependency on government funding, respondents were asked to indicate what percentage of the CSO’s budget originated from government subsidies in the past year. This resulted in a right-skewed distribution. Considering that the survey question was intended to serve as an ordinal measure, a categorical variable was created: no funding (*n *= 1118), government funding contributing 0.01–50% of the budget (*n *= 498) and government funding contributing 51–100% of the budget (*n *= 445).

In addition to country of origin (included as dummies), two other control variables were included. First, we categorised CSOs into business associations (*n *= 579), professional associations (*n *= 400), labour unions (*n *= 66), identity groups (*n *= 263), cause groups (*n *= 456), leisure associations (*n *= 211) and associations of institutions and (semi-)public authorities (*n *= 86). An alternative measure capturing the size of CSOs and distinguishing between individual and organisational members is operationalised in the Appendix. Second, age was added; older CSOs are more likely to be established organisations with high functional differentiation (Rucht 1999). The log-transformed age variable has a minimum of 0.10, a mean of 3.27 (*α* = 0.93) and a maximum of 7.61.

## Analysis

We present a bivariate analysis of the relationship between internal decision-making modes and membership influence. Table 1 shows the distribution of membership influence on CSO policy positions by mode of internal decision-making. Roughly speaking, we can distinguish among three types of CSOs. First are CSOs in which membership is strongly symbolic and in which the staff decide on political positioning; 9.5% of our sample belongs to this category. Hence, few CSOs corresponded to the model of ‘check book members’ lacking decision-making rights (see Jordan and Maloney 2007, p. 127; McCarthy and Zald 1973, p. 20). Indeed, membership influence was the lowest in these organisations (in this category only 22% had ‘very influential’ members). An example of a CSO with a symbolic membership is the Belgian consumer organisation Test-Aankoop/Achats, which is a founding member of the European Consumer Organisation (BEUC) and employs more than 400 staff members. The role of the members is reduced to that of customers. This is, ironically, illustrated by a 2013 case in which the Belgian Jury of Ethical Practices in Advertisement judged that Test-Aankoop had misled its members. The consumer association recruited new members by offering a free ‘tablet’, which was actually an inferior gadget (Depuydt 2016).

**Table 1:** Membership influence by modes of decision-making (*n *= 2122)

	Not/somewhat influential (%)	Moderately influential (%)	Very influential (%)	Total percentage of CSOs (%)
Members: consensus	8.6	31.9	59.4	30.9
Members: voting	10.1	35.0	54.9	
Board: consensus	17.9	46.6	35.4	59.6
Board: voting	17.5	46.0	36.5	
Senior staff	28.4	49.5	22.2	9.5
Total	16.4	42.6	41.0	100

Second, members have more influence on policy positions if the board decides by consensus (35% are ‘very influential’) or through voting (36% are ‘very influential’)—which were modes of decision-making in 60% of the CSOs. In these CSOs, members had a representative role—for instance, the board was usually composed of (important) members elected by the general assembly. An example is the Association of the Dutch Chemical Industry (VNCI). The board is composed of prominent companies and the presidents of other sector associations (e.g. soap producers) who are elected by the members of the VNCI general assembly; the board in turn authorises the organisational leadership and holds those individuals accountable. Another example is the Red Cross in Flanders. That organisation employs more than 1300 staff members, while volunteers hold voting rights at the highest organisational levels (e.g. direction committee of humanitarian services).

Third, if members are formally entitled to decide on policy positions, either by consensus or by voting, they have substantial influence (60% and 55% are ‘very influential’, respectively). In these CSOs, members play an active role, and the membership is highly involved in political positioning. An example is the Association of Free Trade Unions of Slovenia, which is comprised of 22 sectorial unions and an extensive network of regional offices. Decision-making in trade unions is usually aimed at securing mass support among members and at incorporating and aligning different factions. Trade unions often closely engage their members in advocacy work, mass demonstrations and strikes. In sum, the descriptive results indicate that CSOs such as Test-Aankoop are an exception; our data demonstrate that in most cases, members play a representative or active role.

Table 2 presents the ordered logistic regression models analysing the likelihood of members being influential in establishing policy positions. The dependent variable captured whether a CSO’s members were (1) ‘not to somewhat influential’, (2) ‘moderately influential’ or (3) ‘very influential’. Numeric variables were standardised by subtracting the mean and dividing the result by two times the standard deviation; this approach facilitated a comparison between the two measures of professionalisation and aided the interpretation of the interaction parameters (Gelman 2008). Moving one unit of analysis corresponds to moving one standard deviation below the mean to one standard deviation above the mean.

**Table 2:** Predicting membership influence (ordinal logistic regression)

	Model I	Model II	Model III
Intercept	**–**	**–**	**–**
1/2	− 1.697	− 1.725	− 1.730
	(0.153)	(0.153)	(0.154)
2/3	0.489	0.476	0.471
	(0.147)	(0.148)	(0.148)
*Control variables*			
Country: Sweden (ref)	–	–	–
Belgium	0.095	0.091	0.090
	(0.132)	(0.132)	(0.132)
EU	0.100	0.088	0.089
	(0.138)	(0.138)	(0.138)
Lithuania	1.118***	1.104***	1.102***
	(0.182)	(0.183)	(0.183)
The Netherlands	0.693***	0.674***	0.674***
	(0.158)	(0.158)	(0.158)
Slovenia	− 0.285	− 0.302	− 0.303
	(0.203)	(0.203)	(0.203)
Group type Business (ref)	–	–	–
Professional	− 0.197	− 0.216	− 0.217
	(0.133)	(0.133)	(0.133)
Labour	− 0.033	− 0.108	− 0.111
	(0.263)	(0.265)	(0.265)
Identity	− 0.293*	− 0.315**	− 0.319**
	(0.157)	(0.157)	(0.158)
Cause	− 0.469***	− 0.481***	− 0.481***
	(0.136)	(0.136)	(0.136)
Leisure	− 0.296*	− 0.320*	− 0.322*
	(0.167)	(0.167)	(0.167)
Institutions	− 0.129	− 0.157	− 0.159
	(0.231)	(0.232)	(0.232)
Age (log)	0.044	0.036	0.038
	(0.096)	(0.096)	(0.097)
*Main variables*			
Number of staff (log)	− 0.269**	− 0.237**	− 0.235**
	(0.108)	(0.109)	(0.109)
Functional differentiation (ref.cat. = no working groups)	0.123	0.106	0.103
	(0.100)	(0.100)	(0.101)
Membership involvement (index)	0.954***	0.993***	1.055***
	(0.094)	(0.096)	(0.155)
Government funding (ref.cat. = no subsidies)	**–**	–	–
0.01–50% of the budget	− 0.198*	− 0.193*	− 0.192*
	(0.109)	(0.110)	(0.110)
51–100% of the budget	0.062	0.072	0.073
	(0.124)	(0.125)	(0.125)
Political insider (index)	0.322***	0.299***	0.297***
	(0.100)	(0.101)	(0.101)
*Interaction effects*			
Staff * membership involvement	**–**	0.773***	0.787***
		(0.183)	(0.185)
F. differentiation * membership involvement	**–**	–	− 0.096
			(0.187)
Log likelihood	− 1993.373	− 1984.147	− 1984.016
Df	20	21	22
AIC	4026.745	4010.294	4012.031
N	2061	2061	2061

*Note*: *p < 0.1; **p < 0.05; ***p < 0.01; standard errors within parentheses

Model 1 tested the direct effects of the independent variables and included the control variables. With respect to organisation type, the results indicate that members of professional associations, labour groups and groups with institutional members do not differ much from members of business associations in terms of membership influence. In contrast, identity groups, cause groups and leisure groups have less influence compared to members of business associations. One could argue that these results were affected by membership type (corporate versus individual members) and/or the size of the membership body. As these variables strongly overlap with group type—for example, business groups have a corporate membership—we did not include membership variables in models controlling for group type. Table A2 in the Appendix provides alternative models with membership type and size; these models demonstrate that members are considered less influential in groups with individual members. With regard to age, members of older organisations are not more likely to have substantial influence over policy positions. Finally, regarding country differences, the results are in line with Schmitter and Streeck (1999); members are more likely to be influential in more pluralist countries such as Lithuania than in Sweden, which has pronounced neo-corporatist features.

Model I shows that hiring more professionals leads to less membership influence. For every one-unit increase in staff size, the odds of having more influential members (‘very influential’ or ‘moderately influential’ versus ‘not or somewhat influential’) are 0.76 times lower (i.e. 76% *decrease in the odds*), holding all other variables constant. This means that at first glance, one of the basic premises of much research on professionalisation and Hypothesis 1—namely that membership influence declines in organisations with more staff—seems to hold.

**Fig. 1:**
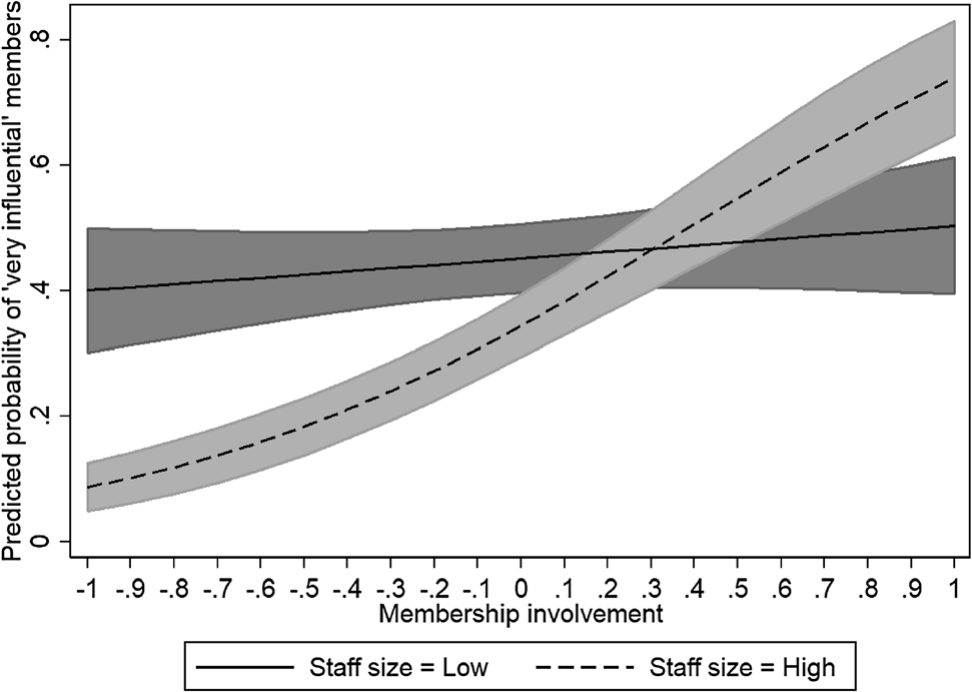
Predicted probabilities for the effect of staff * membership involvement on having “very influential” members

However, this is a partial explanation, as our second hypothesis—that the effect of staff size varies according to the level of membership involvement—was confirmed by Model 2. A high degree of membership involvement combined with more staff is associated with more membership influence. Figure 1 presents how the predicted probabilities of a CSO featuring the highest level of membership influence changes for the highest level of membership influence (*Y*-axis) depending on the degree of membership involvement (*X*-axis; ranging from ‘−1 = one standard deviation below the mean’ to ‘1 = one standard deviation above the mean’) for different staff sizes. Figure A1 (Appendix) presents the marginal effects. For CSOs with much staff, the predicted probability of having ‘very influential’ members increases by 33% when moving from low membership to extensive membership involvement. No such effect is observed for CSOs with small staff sizes. In short, hiring more staff is not invariably associated with less membership influence. To the contrary, combining strong membership involvement and sufficient staff can foster an organisational context in which members can be influential. Professionals can consciously enable involvement—and thus membership influence—when deciding on the organisation’s policy position, which in turn results in a stronger claim on policymakers’ attention.

With regard to functional differentiation, Model 1 indicates that this variable  does not affect membership influence. Model 3 also demonstrates that membership involvement does not moderate the impact of functional differentiation. Alternatively, we tested a model (Table A2, Appendix) with decision-making mode—whether staff, professionals or members make decisions on policy positioning—as an independent variable. Interestingly, the interaction term confirms that professional staff can facilitate membership influence, contingent on functional differentiation.

Political insiderness, contrary to our expectations formulated in Hypothesis 3, has a positive effect on membership influence. For every one-unit increase in insiderness, the odds of having more influential members (‘very’ or ‘moderately influential’ versus ‘not or somewhat influential’) is multiplied 1.38 times (i.e. 38% *increase in the odds*), holding constant all other variables. These findings can be understood within the broader context of CSOs that claim to be representative to secure access to the policymaking process (Albareda and Braun 2019; Grömping and Halpin 2019). Policymakers often need (broad) societal and political support, which incentivises CSOs to closely engage their members in political positioning.

Our analysis of Hypothesis 4 demonstrates that the percentage of the budget coming from government funding is significantly related to membership influence, albeit this effect is only present for CSOs that receive ≤ 50% of their budget from government funding. While many CSO studies have confirmed the impact of government funding on advocacy work (e.g. moderating policy positions, inducing a shift towards service-delivery, affecting the targeting of policymakers), receiving high levels of funding (> 50%) does not seem to affect members’ influence on political positioning (Bloodgood and Tremblay-Boire 2016; Mosley 2012; Neumayr et al. 2015). Hence, we have to  be cautious when assessing our fourth hypothesis, which reasons that the more CSOs depend on government funding, the lower the degree of membership influence. An alternative model with the percentage of the budget obtained from membership fees demonstrates that the more CSOs financially depend on members, the more influence members have on political positioning (Table A3, Appendix). Many CSOs have not cut their financial ties with their members; membership fees, on average, still comprise 52% of their budgets. Again, this result confirms the relevance of strong membership ties for many CSOs.

## Conclusion

This article started from the observation that CSOs differ strongly in the extent to which their members can influence policy positioning (Binderkrantz 2009). CSOs face problems at both ends of the membership influence spectrum. On the one hand, if members have no influence and CSOs are predominantly staff-led, CSOs might risk losing their support base and lack credibility in the eyes of policymakers. On the other hand, strong membership influence may curtail the discretion and autonomy of the organisational leadership. In this case, CSOs risk internal deadlock and might have less flexibility and political effectiveness (Beyers 2008; Grömping and Halpin 2019; Greenwood 2002; Schmitter and Streeck 1999). This study sought to explain the varying levels of membership influence. Scholarship on this subject dates back to the influential work of Michels (1915) and spans various sub-disciplines. Despite the prominence of this topic in the literature, few researchers have analysed the relationship between professionalisation and membership influence in a systematic fashion, across countries and for a wide range of organisations (for an exception, see Binderkrantz 2009). Nevertheless, researchers often take for granted that professionalisation invariably leads to less membership influence (Eikenberry and Kluver 2004; Jordan and Maloney 1997, 2007; Maier et al. 2016; Skocpol 2004). This presumption is inconsistent with recent work beginning to sketch a different theoretical and empirical image (Albareda and Braun 2019; Binderkrantz 2009; Diefenbach 2019; Grömping and Halpin 2019).

Our results further qualify the pessimistic accounts of the relationship between professionalisation and membership influence. First, at least in the case of the surveyed organisations, membership influence is not in complete disarray; our analysis revealed considerable variation in membership influence across various organisation types. Although 81% of all CSOs employ professional staff, staff members are formally entitled to decide on policy positions in only 9% of CSOs. Only a small subset of CSOs correspond to the so-called ‘protest business’ in which members have few or no decision-making rights (Jordan and Maloney 1997, 2007). Our results confirm that many CSOs are governed by rules and decision-making procedures that introduce checks and balances for not only members but also staff and/or organisational leaders (Diefenbach 2019, p. 10).

Second, hiring staff does not always decrease membership influence, as previously claimed (e.g. Jordan and Maloney 1997; Michels 1915; Skocpol 2004). In fact, CSOs that combine strong membership involvement with professional staff positively contribute to membership influence. These findings have important implications for CSO management. To facilitate membership influence, organisations must invest in a robust organisational structure and recruit professionals. Or, in the words of Staggenborg, who was focussed on social movement organisations, ‘Based on my data, I dispute the conclusion that formalised SMOs [social movement organisations] necessarily become oligarchical. In fact, many seem more democratic than informal SMOs because they follow routinized procedures that make it more difficult for individual leaders to attain disproportionate power’ (1988, p. 604).

Furthermore, the analysis clarified the relationship between external dependencies and membership influence. Previously, scholars had suggested that CSOs that are strongly connected to the political system have less influential members (Greenwood 2002; Schmitter and Streeck 1999). However, if CSOs regularly supply information to policymakers, participate in consultations and/or have a seat in advisory councils—in short, if they become political insiders—membership influence increases. Policymakers not only demand expertise but also often need political support. In this regard, CSOs that enjoy strong backing from their members are more representative, and policymakers see them as more legitimate (Bouwen 2002, p. 370; see also Albareda and Braun 2019; Grömping and Halpin 2019). Political insiders are also frequently involved in policy implementation. Smooth implementation is achieved when organisational leaders have considered and aligned the individual concerns of their members since doing so ‘tends to increase the acceptance of regulation by those affected by it’ (Streeck and Schmitter 1985, p. 132). Moreover, while government funding itself shows no unambiguous effect on membership influence, a strong dependence on membership fees is associated with greater membership influence. In short, the results on external conditions further confirm the persistent importance of members.

In conclusion, professionalisation does not always lead to less membership influence. The overall set of CSOs from six political systems displayed substantial variation in membership influence. CSOs that closely involve members in political positioning and that also enjoy substantial professional support might do more good than harm. Moreover, the organisational setup and procedures can guide the behaviour of professionals and mitigate staff influence. Our results confirm that these factors merit future scholarly attention. Instead of being solely staff-led, many CSOs are characterised by a dedicated and influential membership.

## Electronic supplementary material

Below is the link to the electronic supplementary material.

Supplementary material 1 (DOCX 124 kb)

## Data Availability

Replication materials are available in the dataverse-account of the corresponding author at https://dataverse.harvard.eu/.
