# Supplementary material for: Do Professionals Take Over? Professionalisation and Membership Influence in Civil Society Organisations
Source: Voluntas. 2020 Mar 24;31(6):1226–38. doi: 10.1007/s11266-020-00214-9 (PMC7679303; doi:10.1007/s11266-020-00214-9)
Supplement: Supplementary file 1 — Supplementary material 1 (DOCX 124 kb) [file 11266_2020_214_MOESM1_ESM.docx]

**Appendix**

Do Professionals Take Over?

Professionalisation and Membership Influence in Civil Society Organisations

The first part of this appendix is based on

Beyers, J., D. Fink-Hafner, W. Maloney, M. Novak, and F. Heylen (2020). The Comparative Interest Group Survey Project: Project design, practical lessons, and datasets. *Interest Groups & Advocacy*. doi: 10.1057/s41309-020-00082-0

[www.cigsurvey.eu](http://www.cigsurvey.eu)

**Table of content**

[1 Overview of sampling procedures and survey methods 1](#_Toc35262729)

[1.1 Mapping CSO communities and sampling CSOs 1](#_Toc35262730)

[1.2 Overall survey plan 3](#_Toc35262731)

[1.2.1 Identifying respondents 4](#_Toc35262732)

[1.2.2 Approaching and incentivising respondents 5](#_Toc35262733)

[2 Post-estimation: marginal effects plots 7](#_Toc35262734)

[3 Operationalisation of alternative variables and regression models 8](#_Toc35262735)

[3.1 Membership type and size 8](#_Toc35262736)

[3.2 Modes of decision-making 8](#_Toc35262737)

[3.3 Membership fees 8](#_Toc35262738)

[4 References 11](#_Toc35262739)

# **Overview of sampling procedures and survey methods**

Analysing the management, political strategies and organisational development of CSOs is vital to get a better understanding of systems of interest representation. This appendix provides an overview of the overall set-up of our survey project. More precisely, we clarify the different methods of sampling CSOs, how we identify and characterise national communities of CSOs, the survey plan and recruitment of organisational leaders as respondents. We also give an overview of the datasets that are publicly available via a project-website.

Our survey-project is an offspring of another European-wide collaborative project that entailed the mapping and surveying of EU-level interest associations (see project-website). Building on this successful experience we decided to strengthen the comparative leverage of the project by replicating it in several European countries, and hence a large organisational survey project was launched. The core of the project is funded by grants from the Research Foundation-Flanders (FWO-V), the Slovenian Research Agency, and the European Research Council (ERC); separate national CIG-surveys depended on grants from national research agencies.

The overarching aim was to collect systematic data on various organisational aspects of CSOs in a wide range of European political systems. We conducted 10 country online-surveys, in Belgium, The Netherlands, Sweden, Lithuania, Slovenia, Spain, Portugal, Italy, Poland, Czech Republic, and at the European Union (EU) level. The Czech, Italian, Portuguese and Spanish data are not yet available for comparative analysis. In total more than 15,000 organisations were invited to participate, almost 5000 completed the questionnaire with an average response rate of 36%. As this article focuses only on six political systems for which we approached 9850 CSOs, our master data-sets consists of 3732 valid responses (i.e. organisations that answered ≥50% questions). Due to item non-response on several variables, a final set of 2061 observations is included in the analyses.

## **Mapping CSO communities and sampling CSOs**

In contrast to other survey research (e.g., public opinion), where registers of citizens are usually available, comprehensive registers of organisational populations are rare. This means that we had to invest a significant amount of time in constructing a representative list of CSOs. Furthermore, the absence of reliable registries makes the use of standard sampling techniques like stratified or random sampling difficult. In addition to this, the limited systematic mapping of interest intermediation systems in many countries means that studies on CSOs, in contrast to those on parties, government formation, legislative or electoral politics, often lack sound comparative empirical links to the way societal interests are represented via organised interests (see Halpin and Jordan 2011 for an overview in this area). This is accentuated in the case of smaller and Central and Eastern European countries.

A continuing challenge facing researchers involves the conceptual ambiguity that surrounds the main concept (Beyers et al. 2008; Jordan et al. 2004). Defining concepts such as an ‘interest organisation’ or ‘interest community’ or ‘civil society organisation’ is not straightforward. Although different definitions and conceptualisations are not in themselves a problem, scholars are often unclear about which kind of organisations are included or excluded in an appropriate sample. Conceptual clarity (or lack of thereof) has important consequences for the interpretation of research results and generalisability.

One commonly used definition includes three criteria: (1) being organised, (2) aiming to influence public policy, and (3) achieving political objectives through informal and formal political engagements outside the electoral arena (Beyers et al. 2008; see also Andrews and Edwards 2004; Jordan et al. 2004). The latter component sets CSOs apart from political parties; typically, CSOs do not seek office through elections, but try to achieve their goals through formal (e.g. advisory councils) or informal engagements with policymakers. The first component refers to the fact that a minimal level of organisation is required, thus excluding broad movements, waves of public opinion and/or the political activities of individual citizens. Finally, these organisations should show some level of political activity or articulate a public policy interest, which implies that they aim to influence policy outcomes.

Importantly, many politically active organisations are not established with the aim to represent some interest, cause or constituency, but rather for other purposes (such as producing cars, governing a territory, providing education, etcetera). Political representation is thus a by-product of their core business; if their interests are threatened they mobilise politically. Often, the expertise such actors have at their disposal, as well as their economic significance (in terms of employment and/or investments), means they play a crucial role in any political system (Salisbury 1984; see also Lowery 2007). Yet, individual companies, firms, hospitals or universities have no intermediary function, they do not represent a constituency or member base, and their potential political activities are in most cases largely self-regarding. As our project aimed to develop a better understanding of the tensions political organisations experience between, survival, political insiderness and responsiveness to their members/supporters and goals, we decided not to include these so-called ‘pressure participants’ in the survey (Jordan et al. 2004).

Depending on which conceptual component – organizing for collective action or political advocacy – is emphasised, scholars tend to apply two kinds of data collection strategies for mapping organisational populations, focusing either on behavioural (advocacy or lobbying for policy influence) or organisational aspects (mobilising a constituency) (Berkhout et al., 2018). Scholars studying policymaking processes are inclined to adopt a top-down mapping strategy using registers of relevant organisations (for examples see Baumgartner et al. 2009; Beyers et al. 2014; Dür et al. 2019; Fraussen et al. 2015; Hanegraaff et al. 2015; Klüver 2012). Although a behavioural top-down approach is well-suited for research on interest organisations’ policymaking participation, it is somewhat less suitable for studying how organised interests act as intermediaries between society and the state. More concretely, our surveys focus on both membership organisations or ‘groups of’ and non-membership or ‘solidarity organisations’, the so-called ‘groups for’. These solidarity organisations can be divided between primarily politically active cause organisations (like Greenpeace, World Wildlife Fund and the European Anti-Poverty Network) and primarily service providing organisations (Red Cross, Doctors without Borders). Many of these latter groups that represent a collective constituency interests or cause can be labelled as ‘latent interest organisations’ as they are politically less visible and/or active. However, the fact that they often provide important public goods, makes them politically relevant.

Accordingly, we relied largely on bottom-up mapping and different country surveys relied on directories, registries or encyclopaedias of organisations that list all the associations in a particular political system (see Table A1). Most of the sources used had no threshold in terms of political activities; all organisations were included. Some national surveys used a top-down mapping that may approximate to the results of a bottom-up approach as the threshold for political activity is very low. For instance, in the Swedish case, colleagues used a database with all associations that had sent one letter to a government agency in a time span of several years (and combined this with other sources). In short, we deliberately sought to include constituency-based organisations, including those that exhibit lower or less visible levels of political activity. Making valid claims about balancing organisational maintenance, constituencies’ interests, and engaging with policymakers would be rendered impossible if one of the main outcomes (rarely or seldom being politically active) was systematically excluded. Second, empirically, some problems would have arisen when determining a specific cut-off point of political activity, which is further complicated when trying to make this assessment based on the website of an organisation.

## **Overall survey plan**

Constructing a well-defined survey plan is critical for three main reasons: i) to obtain a viable response rate –, Marchetti (2015) estimated an average response rate of 41% in interest organisation surveys; ii) to reduce survey bias due to a lack of response from specific respondents (Dillman et al. 2014); and iii) to combat survey-fatigue – many CSOs are frequently invited by researchers and policymakers to take part in various research projects. One of the unique features of our project is that highly similar surveys were undertaken in various European countries. This entailed establishing a coherent and equivalent approach within each country taking into account local circumstances. We cannot report in detail on each separate survey (see [www.cigsurvey.eu](http://www.cigsurvey.eu) for an exhaustive overview of how national surveys were conducted). Here we sketch the general contours of the overall survey plan which entails developing an appropriate questionnaire, identifying respondents within CSOs and detailing how to approach and motivate these respondents.

We developed a core-questionnaire that included supplementary space for country specific questions relevant to different national contexts. The core-questionnaire was piloted among a small group of respondents in Belgium, the EU, Slovenia and the Netherlands. Following these pilots, we refined and tightened the questionnaire (see Beyers et al. 2020 for further details on this procedure).

### **Identifying respondents**

CSO representatives are often embedded in a professional environment that is internally differentiated. Many, especially larger, organisations have a front office, a number of policy experts (in some cases organised in working groups/committees), a secretary general, and a board of directors headed by a president supported by auxiliary staff, interns and volunteers. Furthermore, large associations are often clusters of organisations, headed by a (national) office and subdivided by regional/local chapters. In such organisations, it is not always easy to locate the most appropriate respondent and sometimes it is unlikely that one person will be able to answer a wide variety of questions. In contrast, smaller organisations, especially those located in consolidating democracies, tend to not employ professional staff. Organisational complexity and diversity is a critical consideration when seeking to identify potential respondents.

It is crucial to personalise communications – i.e. try to avoid sending invitation letters to a general email-address. Personalisation can engender ties and trust between the research team and the respondents (Dillman et al., 2014; Cook et al. 2000; Cycyota and Harrison, 2002). Accordingly, in each case we identified an organisational official or spokesperson (e.g. the chairperson, the director, the secretary-general). In most settings, it is considered polite and courteous to use full names and titles when approaching potential respondents. It also demonstrates that the research team has done its homework and due diligence on basic organisational facts before inviting experts to take part in a survey.

The different national survey teams invested a significant amount of time creating lists with names of key spokespersons and in most cases it was possible to identify at least one person (e.g. chairperson, or director) as well as their email address. A small number of cases – less than one percent – for which we could not identify an individual or a general info address were dropped from the sample. For large organisations we attempted to identify two individuals (e.g., the president and the secretary general). Efforts to identify key spokespersons included website searches and telephone calls to the organisations (if such information was not available on the website). A personalised approach required collecting evidence on the name of the organisation, abbreviation, full name of the respondent, email address, telephone number, and gender. Great care was taken when collecting contact information as errors in gender, the related grammatical errors or spelling mistakes may invariably lead to non-responses.

### **Approaching and incentivising respondents**

It is crucial to have a thorough plan on how to approach and incentivise respondents. In contacting respondents, the survey teams adopted various techniques aimed at reducing barriers and positively influencing respondent’s willingness to take part in the survey (Cycyota and Harrison 2006; Frohlich 2002). The most important aspect here is the careful planning and implementation of follow-up reminders via e-mail and/or telephone (Roth and Be Vier 1998). Experimental research shows that repeated contacts signal the legitimacy of a survey project and the willingness of the researcher to invest time and resources in reaching out to respondents (Sauermann and Roach 2013). Initial invitation letters help to establish trust by providing contact details such as mail addresses, telephone numbers and the project-website. Several national surveys used postal letters (with university logos) as the initial form of communication. Sending personalised pre-notification letters might make a survey stand out and shows the commitment of the research team; especially if the letter is signed by the project leader. Each national survey adopted procedures involving repeated interactions with respondents and this led to substantial improvements in response rates. However, we limited our reminder contacts to four (via email and/or telephone) in order to avoid being seen as ‘pushy’. The final reminder included a closing date. Our exchanges with our respondents were sensitive to the varying national contexts. For instance, the Slovenian invitation letter stressed that this was a major international project, while the Polish project emphasised that the survey was being led by researchers based at a Polish university. Finally, we incentivised our respondents in a variety of ways, in the Belgium and EU surveys the research teams made a donation to a charity for each completed survey, other incentives included a report on preliminary research results and/or an invitation to the presentation of the first results.

|  |  | Mapping sources | Sample size | Response rate | Time fieldwork | Principal research team |
| --- | --- | --- | --- | --- | --- | --- |
| 1 | EU-level | - Transparency Register - OECKL - Top-down mapping INTEREURO | 2037 | 754 (37%) | March 2015 – June 2015 | Jan Beyers, University of Antwerp |
| 2 | Belgium | - Crosspoint Bank for Enterprises - Sectorlink - Filantropie.be | 1687 | 759 (45%) | January - May 2016 | Jan Beyers, University of Antwerp |
| 3 | Slovenia | - Agency of the Republic of Slovenia for Public Legal Records and Related Services (AJPES) | 1203 | 439 (36%) | November 2015 - February 2016 | Danica Fink-Hafner, University of Ljubljana |
| 4 | Netherlands | - Public Hearing Dutch House of Representatives - Pyttersen’s Almanak | 2479 | 768 (31%) | April 2016 – December 2016 | Marcel Hanegraaff and Joost Berkhout, University of Amsterdam |
| 5 | Sweden | - Publicly available mail-exchanges between government and organized interests (government archives) - Responses to public consultations - Groups that organized events during Almedalsveckan (in 2011) - Groups identified in the INTEREURO-project | 1540 | 647 (42%) | June 2015 - September 2015 | Daniel Naurin, University of Gothenburg |
| 6 | Lithuania | - The directory of Lithuanian business entities - List of organizations provided by ten Lithuanian Ministries - Interest groups known to the research team as active in the public policy process | 904 | 365 (40%) | September 2016 – November 2016 | Ligita Šarkutė and Algis Krupavičius, Vytautas Magnus University |
|  | TOTAL |  | 9850 | 3732 (36%) |  |  |

**Table 1. Overview of datasets**

# **Post-estimation: marginal effects plots**

The marginal effects plots are based on Model 2 (main text) and allow to interpret for which combinations of values the interaction significantly differs. Figure A1 indicates the predicted difference in membership influence (Y-axis) for different degrees of membership involvement (X-axis). Marginal effects are shown for each outcome of the dependent variable. Figure A1, as expected, shows that a unit increase in the value of staff size, on average, leads to a decrease in the probability of the dependent variable to take the value of one (i.e. lowly influential) if members become more involved. Vice versa, a unit increase in the value of staff size, on average, leads to an increase in the probability of the dependent variable to take the value of three (i.e. highly influential) if members become more involved. Differences in membership influence are significant for higher levels of membership involvement.

**Figure A1. Average marginal effects of staff size across degrees of membership involvement with 95% CIs**


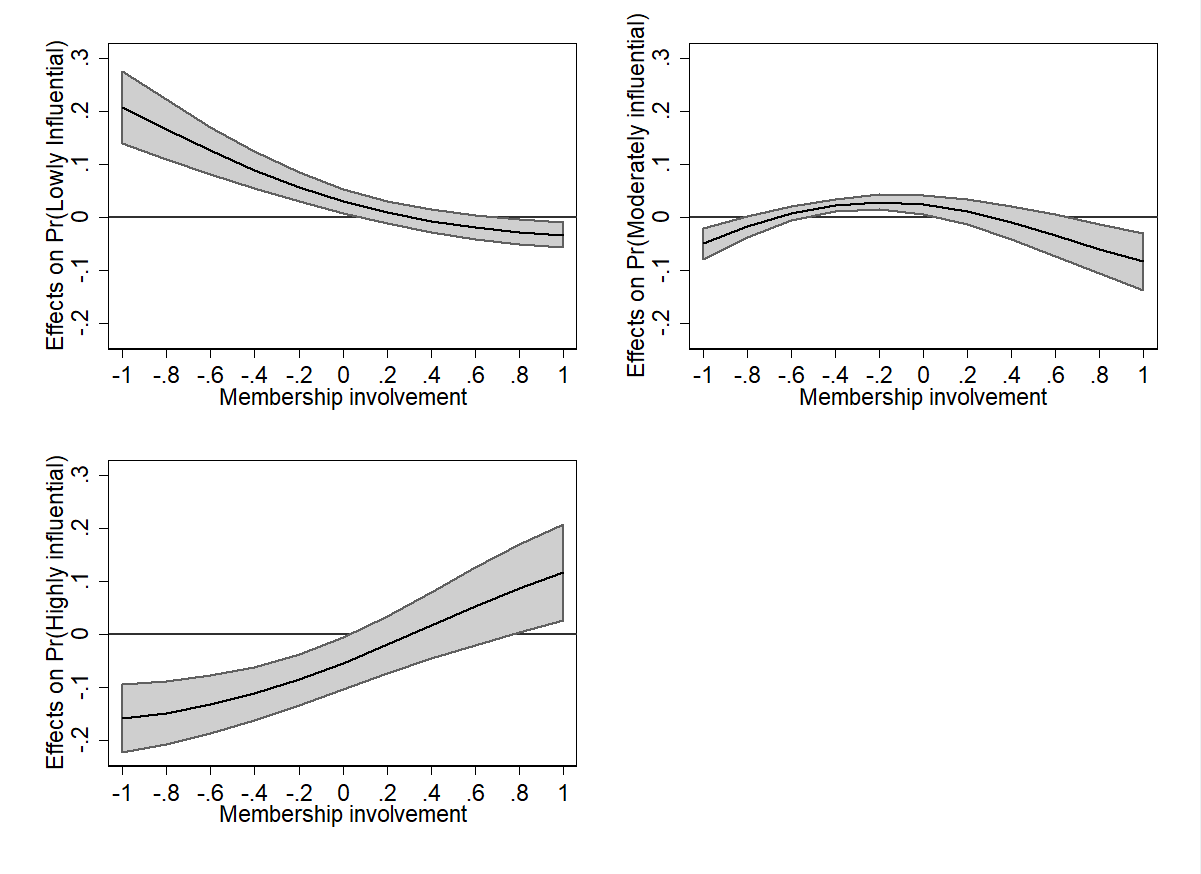


# **Operationalisation of alternative variables and regression models**

## **Membership type and size**

Membership influence might be linked to membership size. For large membership organisations, it is more difficult for the leadership to include and listen to all members during internal decision-making processes, while members themselves face collective action problems when trying to shape policy positions (De Bruycker et al. 2019; Schmitter and Streeck 1999: p. 25). Therefore, we control for two measures of membership size, one for individual and one for organisational membership. This distinction is important, as one cannot directly compare the individual membership of labour unions with the corporate membership of business associations. Due to the skewedness of these distributions and the survey question being intended as an ordinal question, two measures were created with three categories for both individual membership and organisational membership respectively: (1) below the average number of individuals (n=967) or organisations (n=837), (2) the average number of individuals (n=348) or organisations (n=418), and (3) above the average number of individuals (n=676) or organisations (n=736).

## **Modes of decision-making**

Many CSOs have formal rules with regard to how decisions are made, and these rules are likely to impact membership influence. The following question was asked: “*Decisions can be made according to different modes, for example via consensus or voting. How does your organisation take decisions?”.* Respondents could pick among the following categories: ‘consensus among members’, ‘voting among members’, ‘consensus in the board’, ‘voting in the board’, ‘senior staff decides’. These responses were simplified to create a categorical variable distinguishing between (1) members decide (n=617), (2) board decides (n=1187), and (3) staff decides (n=187).

## **Membership fees**

Dependence on membership fees, was measured as the percentage of the budget originating from membership fees in the past year. This variable has a distribution with a minimum of 0%, a mean of 51.4% (α=39.95) and a maximum of 100%.

**Table A2. Models with membership type, membership size, and decision-making mode**

|  | **Model I** | **Model II** | **Model III** |
| --- | --- | --- | --- |
| Intercept | **-** | **-** | **-** |
| 1/2 | -2.452 | -2.506 | -2.614 |
|  | (0.175) | (0.177) | (0.207) |
| 2/3 | -0.242 | -0.293 | -0.397 |
|  | (0.166) | (0.168) | (0.199) |
| CONTROL VARIABLES |  |  |  |
| Country: Sweden (ref) | - | - | - |
| Belgium | -0.102 | -0.114 | -0.113 |
|  | (0.127) | (0.128) | (0.128) |
| EU | 0.032 | 0.028 | 0.026 |
|  | (0.138) | (0.138) | (0.138) |
| Lithuania | 1.173*** | 1.159*** | 1.149*** |
|  | (0.182) | (0.183) | (0.183) |
| Netherlands | 0.800*** | 0.810*** | 0.819*** |
|  | (0.160) | (0.160) | (0.160) |
| Slovenia | -0.286 | -0.240 | -0.232 |
|  | (0.206) | (0.208) | (0.208) |
| Number of individual members (ref=none) | - | - | - |
| N individual members (below average) | -0.306** | -0.311** | -0.305** |
|  | (0.130) | (0.131) | (0.131) |
| N individual members (above average) | -0.540*** | -0.551*** | -0.546*** |
|  | (0.114) | (0.114) | (0.114) |
| Number of business members (ref=none) | - | - | - |
| N business members (below average) | -0.208* | -0.207* | -0.214* |
|  | (0.122) | (0.122) | (0.122) |
| N business members (above average) | -0.064 | -0.060 | -0.065 |
|  | (0.113) | (0.113) | (0.113) |
| Age (log) | 0.006 | 0.010 | -0.0001 |
|  | (0.099) | (0.099) | (0.099) |
| MAIN VARIABLES |  |  |  |
| Number of staff (log) | -0.084 | 0.293 | 0.337* |
|  | (0.112) | (0.190) | (0.195) |
| Functional differentiation(ref.cat.=no working groups) | 0.250** | 0.240** | 0.088 |
|  | (0.103) | (0.103) | (0.183) |
| Decision made by (ref=members) | **-** | - | - |
| - Decision made by board | -0.889*** | -0.928*** | -1.024*** |
|  | (0.103) | (0.105) | (0.183) |
| - Decision made by staff | -1.542*** | -1.594*** | -1.982*** |
|  | (0.167) | (0.178) | (0.276) |
| Government funding (ref=no subsidies) | **-** | - | - |
| - 0.01%-50% of the budget | -0.068 | -0.069 | -0.071 |
|  | (0.111) | (0.111) | (0.111) |
| - 51%-100% of the budget | -0.004 | -0.011 | -0.007 |
|  | (0.118) | (0.119) | (0.119) |
| Political insider (index) | 0.502*** | 0.497*** | 0.497*** |
|  | (0.100) | (0.101) | (0.101) |
| INTERACTION EFFECTS |  |  |  |
| Staff*Decision-making mode |  | - | - |
| - Staff*Board |  | -0.538*** | -0.574*** |
|  |  | (0.208) | (0.214) |
| - Staff*Staff |  | -0.317 | -0.410 |
|  |  | (0.337) | (0.342) |
| F. differentiation* Decision-making mode |  | - | - |
| - F. differentiation*Board |  |  | 0.138 |
|  |  |  | (0.217) |
| - F. differentiation*Staff |  |  | 0.630* |
|  |  |  | (0.339) |
| Log Likelihood | -1905.869 | -1902.482 | -1900.727 |
| Df | 19 | 21 | 23 |
| AIC | 3849.738 | 3846.963 | 3847.454 |
| N | 1,991 | 1,991 | 1,991 |
| *Note: *p<0.1; **p<0.05; ***p<0.01; standard errors in parentheses* | | | |

**Table A3. Models with dependence on membership fees**

|  | **Model I** | **Model II** | **Model III** |
| --- | --- | --- | --- |
| Intercept | **-** | **-** | **-** |
| 1/2 | -1.393 | -1.430 | -1.436 |
|  | (0.178) | (0.179) | (0.180) |
| 2/3 | 0.794 | 0.773 | 0.767 |
|  | (0.175) | (0.176) | (0.177) |
| CONTROL VARIABLES |  |  |  |
| Country: Sweden (ref) | - | - | - |
| Belgium | 0.142 | 0.140 | 0.139 |
|  | (0.131) | (0.131) | (0.131) |
| EU | 0.064 | 0.055 | 0.056 |
|  | (0.137) | (0.138) | (0.138) |
| Lithuania | 1.126*** | 1.110*** | 1.109*** |
|  | (0.182) | (0.183) | (0.183) |
| Netherlands | 0.667*** | 0.649*** | 0.649*** |
|  | (0.158) | (0.158) | (0.158) |
| Slovenia | -0.265 | -0.284 | -0.285 |
|  | (0.203) | (0.203) | (0.203) |
| Group type Business (ref) | - | - | - |
| Professional | -0.141 | -0.161 | -0.162 |
|  | (0.133) | (0.134) | (0.134) |
| Labour | -0.108 | -0.179 | -0.181 |
|  | (0.264) | (0.265) | (0.265) |
| Identity | -0.103 | -0.127 | -0.131 |
|  | (0.161) | (0.162) | (0.162) |
| Cause | -0.296** | -0.309** | -0.310** |
|  | (0.141) | (0.141) | (0.142) |
| Leisure | -0.178 | -0.203 | -0.205 |
|  | (0.168) | (0.168) | (0.169) |
| Institutions | -0.060 | -0.090 | -0.092 |
|  | (0.230) | (0.230) | (0.231) |
| Age (log) | 0.024 | 0.016 | 0.016 |
|  | (0.096) | (0.096) | (0.096) |
| MAIN VARIABLES |  |  |  |
| Number of staff (log) | -0.203* | -0.173 | -0.172 |
|  | (0.109) | (0.109) | (0.109) |
| Functional differentiation (ref.cat.=no working groups) | 0.106 | 0.091 | 0.088 |
|  | (0.100) | (0.101) | (0.101) |
| Membership involvement (index) | 0.948*** | 0.988*** | 1.030*** |
|  | (0.094) | (0.096) | (0.155) |
| Membership fees | 0.004*** | 0.004*** | 0.004*** |
|  | (0.001) | (0.001) | (0.001) |
| Political insider (index) | 0.297*** | 0.275*** | 0.274*** |
|  | (0.100) | (0.101) | (0.101) |
| INTERACTION EFFECTS |  |  |  |
| Staff*Membership involvement | **-** | 0.762*** | 0.772*** |
|  |  | (0.184) | (0.186) |
| F. differentiation*Membership involvement | **-** | - | -0.064 |
|  |  |  | (0.188) |
| Log Likelihood | -1991.708 | -1982.784 | -1982.726 |
| Df | 19 | 20 | 21 |
| AIC | 4021.416 | 4005.567 | 4007.451 |
| N | 2061 | 2061 | 2061 |
| *Note: *p<0.1; **p<0.05; ***p<0.01; standard errors within parentheses* | | | |

#

# **References**

Andrews, K. T., and Edwards, B. (2004). Advocacy Organizations in the U.S. Political Process. *Annual Review of Sociology* *30*: 479-506.

Baumgartner, F. R., Berry, J. M., Hojnacki, M., Kimball, D. C., Leech, B. L. (2009). *Lobbying and Policy Change: Who Wins, Who Loses, and Why.* Chicago: University of Chicago Press.

Berkhout, J., Beyers, J,, Braun, C,, Hanegraaff, M., and Lowery, D. (2018). Making Inference across Mobilization and Influence Research: Comparing Top-Down and Bottom-Up Mapping of Interest Systems. *Political Studies 66*(1): 43-62.

Beyers, J., Bonafont, L. C., Dür, A., Eising, R., Fink-Hafner, D., Lowery, D., Mahoney, C., Maloney, W. & Naurin, D. 2014. The intereuro project: Logic and structure. *Interest Groups & Advocacy*, 3, 126-140.

Beyers, J., Eising, R. & Maloney, W. 2008. Researching interest group politics in europe and elsewhere: Much we study, little we know? *West European Politics*, 31, 1103-1128.

Cook, C., Heath, F., and Thompson, R. L. (2000). A Meta-Analysis of Response Rates in Web- or Internet-Based Surveys. *Educational & Psychological Measurement 60*(6): 821-836.

Cycyota, C. S., and Harrison, D. A. (2002). Enhancing survey response rates at the executive level: Are employee- or consumer-level techniques effective? *Journal of Management* *28*(2): 151-176.

Cycyota, C. S., and Harrison, D. A. (2006). What (Not) to expect when surveying executives: A meta-analysis of top manager response rates and techniques over time. *Organizational Research Methods* *9*(2): 133-160.

De Bruycker, I., Berkhout, J., and Hanegraaff, M. (2019). The paradox of collective action: Linking interest aggregation and interest articulation in EU legislative lobbying. *Governance 32*(2): 295-312.

Dillman, D. A., Smyth, J. D., and Christian, L. M. (2014). *Internet, Phone, Mail and Mixed-mode Surveys. The Tailored Design Method.* New Jersey: Wiley.

Dür, A., Marshall, D., and Bernhagen, P. (2019). *The Political Influence of Business in the European Union.* Ann Arbor: The University of Michigan Press.

Fraussen, B., Beyers, J., and Donas, T. (2015). The Expanding Core and Varying Degrees of Insiderness. Institutionalized Interest Group Involvement Through Advisory Councils. *Political Studies 63*(3): 569-588.

Frohlich, M. T. (2002). Techniques for improving response rates in OM survey research. *Journal of Operations Management 20*(1): 53-62.

Halpin, D. R., and Jordan, G. (2011). *The Scale of Interest Organization in Democratic Politics. Data and Research Methods*. New York; Palgrave.

Hanegraaff, M., Braun, C., De Bièvre, D., and Beyers, J. (2015). The Domestic and Global Origins of Transnational Advocacy: Explaining Lobbying Presence During WTO Ministerial Conferences. *Comparative Political Studies 48*(12): 1591-1621.

Jordan, G., Halpin, D. R., and Maloney, W. A. (2004) Defining Interests: Disambiguation and the Need for New Directions? *British Journal of Politics and International Relations 6*(2): 195-212.

Klüver, H. (2012). *Lobbying in the European Union: Interest Groups, Lobbying Coalitions, and Policy Change*. Oxford: Oxford University Press.

Marchetti, K. (2015). The use of surveys in interest group research. *Interest Groups & Advocacy 4*(3): 272-282.

Roth, P. L., and Be Vier, C, A, (1998). Response rates in HRM/OB survey research: Norms and correlates, 1990–1994. *Journal of Management 24*(1): 97-117.

Salisbury, R. H. (1984). Interest Representation: The Dominance of Institutions. *American Political Science Review 78*(1): 64-76.

Sauermann, H., and Roach, M. (2013). Increasing web survey response rates in innovation research: An experimental study of static and dynamic contact design features. *Research Policy 42*(1): 273-286.

Schmitter, P. C., and Streeck, W. (1999). The organization of business interests: Studying the associative action of business in advanced industrial societies. *MPIfG Discussion Paper* *99*(1): 1–94.
